# Dry Sores

**Published:** 1867-12

**Authors:** 


DRY SORES, its affinity for organic globules is such that it seems to be attracted to them through the dry scab, moistens it, detaches it, and causes a new and healthful surface to grow beneath it; it has the same delightful, soothing and healing effects when applied to

